# Genome-Wide Analysis of the HECT-Type E3 Ubiquitin Ligase Gene Family in *Nicotiana benthamiana*: Evidence Implicating *NbHECT6* and *NbHECT13* in the Response to Tomato Yellow Leaf Curl Virus Infection

**DOI:** 10.3390/genes16101150

**Published:** 2025-09-27

**Authors:** Jin Shen, Shasha Yu, Fang Ye, Yiming Zhang, Xue Wu, Mengxuan Shi, Gen Zhao, Yang Shen, Zhoufo Lu, Zaihang Yu, Xinyu Li, Xueting Zhong, Zhanqi Wang

**Affiliations:** 1 Zhejiang Provincial Key Laboratory of Biology of Crop Pathogens and Insects, College of Life Sciences, Huzhou University, Huzhou 313000, China; 13572564068@163.com (J.S.); 13885475581@163.com (S.Y.); yyff7894@163.com (F.Y.); 19857291457@163.com (X.W.); 13757548172@163.com (M.S.); sy18157382505@163.com (Y.S.); zhoufo_lu999@163.com (Z.L.); yuzaihang6@163.com (Z.Y.); xinyu01230@163.com (X.L.); zxt@zjhu.edu.cn (X.Z.); 2 Department of Life Sciences, University of Southampton, Southampton SO1671BJ, UK; zym13567290330@163.com; 3 Huzhou Academy of Agricultural Sciences, Huzhou 313000, China; zhaogen818@163.com

**Keywords:** *Nicotiana benthamiana*, HECT gene family, phylogenetic analysis, expression profile, TYLCV infection

## Abstract

**Background:** The ubiquitin–proteasome system plays a critical role in plant antiviral defense, with HECT-type E3 ubiquitin ligases serving as key regulators of protein turnover. To explore the potential involvement of the HECT gene family in host resistance against tomato yellow leaf curl virus (TYLCV), a comprehensive analysis was conducted in *Nicotiana benthamiana*. **Methods:** In this study, the HECT gene family in *N. benthamiana* was systematically investigated using a genome-wide bioinformatic analysis. The potential roles of these genes in the response to TYLCV infection were further examined using a virus-induced gene silencing (VIGS) technique. **Results:** Using a Hidden Markov Model approach, 18 *NbHECT* genes were identified that phylogenetically clustered into four subfamilies with distinct structural features. Chromosomal location and synteny analyses indicated that these genes were unevenly distributed across 11 chromosomes, with 10 instances of segmental duplication identified. Tissue-specific expression profiling demonstrated that 17 *NbHECTs* were constitutively expressed, with Group III members showing the highest expression in reproductive tissues. Following TYLCV infection, *NbHECT6* was significantly downregulated while *NbHECT13* was upregulated in both inoculated and systemic leaves. Functional validation through the VIGS approach revealed that suppression of *NbHECT6* and *NbHECT13* increased host susceptibility, as evidenced by exacerbated symptom severity and enhanced viral DNA accumulation compared to controls. **Conclusions:** These findings establish *NbHECT6* and *NbHECT13* as critical components of the plant antiviral response, providing new insights into ubiquitin-mediated defense mechanisms against geminiviruses.

## 1. Introduction

In higher plants, the life cycle—from seed germination and subsequent growth and development to environmental adaptation—is orchestrated by a series of highly coordinated biological processes [1]. In plant cells, proteins function as central molecular executors that regulate a complex network of biochemical pathways, maintaining core physiological functions and facilitating adaptive responses [2]. However, the functions of proteins are not constant; they must be precisely activated, regulated, and degraded at specific times to accommodate the dynamic growth demands and environmental stresses faced by plants [3,4]. Central to this intricate regulatory network is the ubiquitin–proteasome system (UPS), which has been demonstrated to play a crucial role in plant growth, development, and stress responses [5,6,7,8]. The core mechanism underlying the UPS is ubiquitination, a biochemical process mediated by an enzymatic cascade in which ubiquitin (Ub) molecules are covalently linked through their C-terminus to lysine residues of target proteins [9,10]. Ub-tagged proteins are recognized explicitly by various intracellular enzyme complexes and organelles, such as the 26S proteasome, thereby determining the fate of the target proteins, including their degradation, transport, functional modification, or participation in signaling cascades [6,7,8,9,10].

The ubiquitination process is highly organized and sequentially mediated by three classes of Ub-related enzymes (E1, E2, and E3) [9,10,11,12]. Among these, E3 ligases serve as the primary determinants of substrate specificity in the ubiquitination process. Based on their domain architecture and mechanisms of catalyzing Ub chain formation, E3 ligases are broadly classified into four major categories: HECT (Homologous to E6-AP Carboxyl Terminus) type, RING (Really Interesting New Gene) type, CRL (Cullin-RING Ligase) type, and U-box type [13,14], which facilitate Ub transfer through two basic mechanisms: direct transfer and indirect transfer. HECT-type E3 ligases (HECTs) are mainly involved in the indirect Ub transfer [14,15,16]. These E3 ligases possess a conserved HECT domain, which is typically situated at the C-terminus and consists of approximately 350 amino acid residues [15,16]. This domain can be further divided into two functional subdomains: an N-terminal subdomain responsible for facilitating E2 binding, and a C-terminal subdomain that contains a critical catalytic cysteine residue [14,17,18]. This catalytic cysteine residue is crucial for the formation of the Ub-thioester intermediate, which ultimately enables the transfer of Ub to target proteins [17,19,20]. The distinct catalytic mechanism of HECTs and their pivotal role in mediating Ub chain formation constitute key molecular foundations for the identification, functional characterization, and evolutionary analysis of members within this gene family.

Tomato yellow leaf curl virus (TYLCV), which is a monopartite DNA virus within the family *Geminiviridae*, has spread worldwide rapidly since its initial identification in tomato plants in 1983 [21]. It has caused significant losses in tomato cultivation and has become the predominant viral pathogen, posing a threat to global *Solanaceae* crop production [22,23,24,25]. The TYLCV genome comprises eight genes that encode eight corresponding viral proteins (V1–V3 and C1–C5), which coordinately regulate key stages of viral infection [23,24,25,26]. During the invasion and immune evasion stage, the V1 protein (also known as coat protein, CP) assembles into the viral capsid structure, and V2 facilitates nuclear export of CP and functions as a suppressor to counteract both host antiviral gene silencing [27,28]. Furthermore, the C2 protein can interfere with protein ubiquitination and antagonize the jasmonic acid signaling pathway, therefore promoting viral infection [29,30]. During the replication and proliferation stage, the C1/Rep protein plays a central role in viral DNA replication, recruiting the host replication machinery [31]. Meanwhile, the C3 protein significantly enhances viral replication efficiency through its interactions with host factors [32,33]. During the symptom development process, the V3 protein localizes to the endoplasmic reticulum and the Golgi apparatus, inhibiting cell-to-cell spread of RNA silencing and restricting intercellular movement of the virus [34]. The C4 and C5 proteins serve as the primary determinant of symptom development, suppressing the intercellular spread of RNA silencing and interfering with host defense mechanisms and developmental processes [35,36,37]. Thus, it appears that each protein encoded by TYLCV plays a crucial role in its viral pathogenicity.

*N. benthamiana*, a member of the *Solanaceae* family, is extensively utilized as a model organism in plant virology due to its high susceptibility to a wide range of viruses [38]. To explore the role of HECT E3 ligases in the plant response to TYLCV infection, we conducted a comprehensive analysis of the HECT gene family in *N. benthamiana*. In this study, the HECT gene family in *N. benthamiana* was systematically investigated, and their potential roles in response to TYLCV infection were examined using a virus-induced gene silencing (VIGS) technique. A total of 18 members of the HECT gene family were identified in *N. benthamiana*, and two of them were found to be differentially changed during TYLCV infection. Further VIGS assay revealed that suppressing the expression of *NbHECT6* and *NbHECT13* in *N. benthamiana* enhances host susceptibility to TYLCV. Our findings provide new evidence that *NbHECTs* play a significant role in improving plant resistance to TYLCV infection.

## 2. Materials and Methods

### 2.1. Identification of the HECT Genes in N. benthamiana

The HECT protein sequences of *Arabidopsis thaliana* [39], *Oryza sativa* [40], *Zea mays* [41], *Solanum lycopersicum* [14], *Glycine max* [42], and *Triticum aestivum* [43] were obtained from the Ensembl Plants database (https://plants.ensembl.org/ (accessed on 2 December 2023)) (Table S1) and used to generate an HECT Hidden Markov Model (HMM) using HMMER software (v3.3.2) [44]. The genome sequence of *N. benthamiana* (v2.6.1) was downloaded from the Solanaceae Genomics Network database (https://solgenomics.net/ (accessed on 2 December 2023)) [45] and subjected to HMMER search as described previously [26]. The obtained protein sequences were further analyzed using the online InterPro database (https://www.ebi.ac.uk/interpro/ (accessed on 14 December 2023)) [46] to verify the presence of the conserved HECT domain (IPR000569). The candidate *NbHECT* genes identified in *N. benthamiana* were renamed according to their chromosomal distribution order. The physicochemical properties of NbHECT proteins were analyzed using the Compute pI/MW tool available on the Expasy website (https://www.expasy.org/ (accessed on 16 December 2023)) [47].

### 2.2. Phylogenetic Tree Construction, Exon–Intron Structure, and Promoter Analyses

The full-length sequences of HECT proteins from *N. benthamiana*, *A. thaliana*, *O. sativa*, *Z. mays*, *S. lycopersicum*, *G. max*, and *T. aestivum* (Table S1) were aligned using the MUSCLE algorithm implemented in MEGA 11.0 [48]. A phylogenetic tree was generated using MEGA 11.0, employing the maximum-likelihood (ML) method with the JTT + G model with 1000 bootstrap replicates, as previously described [26]. The exon–intron structural information of *NbHECT* genes was obtained from the *N. benthamiana* genome sequence database (v2.6.1) [45] and subsequently visualized using the online tool Gene Structure Display Server 2.0 (GSDS 2.0) [49]. The 2000 bp promoter sequences of *NbHECT* genes were retrieved from the *N. benthamiana* genome and analyzed using the PlantCARE database (https://bioinformatics.psb.ugent.be/webtools/plantcare/html/ (accessed on 18 April 2024)) [50]. The putative *cis*-acting elements were classified and summarized according to their functional roles and visualized using TBtools-II software (v2.148) [51].

### 2.3. Chromosomal Localization and Collinearity Analyses

The chromosomal locations of *NbHECT* genes were obtained from the GFF3 annotation file of the *N. benthamiana* genome. Collinearity analysis was performed using the MCScanX toolkit (v1.0.0) as described previously [52]. Tandem duplications were identified as genes with no more than five adjacent duplicates located within a 100-kb positional interval on the same chromosome as described previously [53,54]. The non-synonymous (Ka) and synonymous (Ks) substitution rates, along with the corresponding Ka/Ks ratio for each gene pair, were calculated using TBtools-II software (v2.148) [51]. For the evolutionary analysis, the divergence times (T) of duplication events were estimated using the formula T = Ks/2λ, where λ represents a substitution rate, which was set to 1.5 × 10^−8^ for *N. benthamiana* [55,56].

### 2.4. Tissue-Specific Expression Pattern Analysis

To explore the expression profiles of *NbHECT* genes in different tissues of *N. benthamiana*, we performed an integrative comparative analysis using publicly available RNA-sequencing datasets. Eight different tissues, including the roots, stems, leaves, flowers, capsules, apices, calli, and seedlings, under accession numbers, SRR696961, SRR696992, SRR696940, SRR696938, SRR696884, SRR685298, SRR697013, and SRR696988 of NCBI BioProject PRJNA188486 [57], were selected to analyze the expression patterns of *NbHECTs* in *N. benthamiana*. Sequence assembly was conducted using HISAT2 (v.2.1.0) [58], and the transcripts per million (TPM) values were computed using StringTie2 (v.2.1.5) [59]. The results were visualized using TBtools-II software (v2.148) [51].

### 2.5. Plant Materials and TYLCV Inoculation

In this study, wild-type *N. benthamiana* plants were used and were cultivated under controlled conditions in a greenhouse at Huzhou University as previously described [26]. The infectious clone of TYLCV was constructed as described by Zhang et al. [60]. Viral inoculation of *N. benthamiana* with TYLCV was performed as described previously [26,61]. At 6 and 14 days post-infiltration (dpi), inoculated and systemic leaves of *N. benthamiana* plants were sampled, respectively, from three independent biological replicates as described previously [62].

### 2.6. RNA Extraction and Quantitative PCR (qPCR) Analysis

Total RNA was isolated from leaf samples using the FastPure Plant Total RNA Isolation Kit (Vazyme, Nanjing, China), and residual genomic DNA was subsequently removed by DNase I following established protocols [63]. The concentration and integrity of RNA were assessed using an Agilent 2100 Bioanalyzer system (Agilent Technologies, Santa Clara, CA, USA). cDNA synthesis and qPCR analysis were conducted as described previously [64]. Briefly, the cDNA was synthesized from 1 μg of total RNA using the Prime Script^TM^ RT Reagent Kit (TaKaRa, Dalian, China), followed by qPCR conducted on a CFX96 Touch Deep Well Real-Time PCR Detection System (Bio-Rad, Hercules, CA, USA). For each candidate gene, PCR reactions were conducted in duplicate, and relative mRNA expression levels were quantified using the 2^−ΔΔCt^ method [65], with data derived from three independent biological replicates. *N. benthamiana ACTIN2* gene (*NbACTIN2*) was used as an internal control [23,66]. The primers used for qPCR analysis were designed using TBtools-II software (v2.148) [51], and they are listed in Table S2. The results represent the averages of three biological replicates, with raw data provided in Table S3.

### 2.7. VIGS Experiment and Silencing Efficiency Assay

To suppress the expression of the *NbHECT6* and *NbHECT13* genes in *N. benthamiana*, a tobacco rattle virus (TRV)-based VIGS system was employed [67]. To construct the VIGS plasmids, a 300 bp fragment of the *NbHECT6* and *NbHECT13* genes was amplified and subsequently cloned into the EcoRI–BamHI restriction sites of the pTRV2 vector as described by Zhong et al. [68]. The primers used for VIGS plasmid construction are listed in Table S2. *Agrobacterium*-mediated infiltration of *N. benthamiana* was conducted as previously described [64]. *N. benthamiana* plants agroinfiltrated with TRV1 and TRV2:GFP served as the control. At 10 dpi, the silencing efficiency was determined by qPCR analysis, as previously described [26,68]. The primers used are listed in Table S2.

### 2.8. Genomic DNA Extraction and Viral Accumulation Determination

Total genomic DNA was extracted from systemically infected leaves using a cetyltrimethylammonium bromide (CTAB)-based method [66]. The accumulation of viral genomic DNA was assessed by qPCR, and the relative levels were determined using the 2^−ΔΔCt^ method [65]. The *N. benthamiana 25S nuclear rRNA* gene (*Nb25SrRNA*) served as an internal reference [23]. The primers used are listed in Table S2.

### 2.9. Statistical Analysis

All experiments were conducted with three independent replicates, and data are presented as mean ± standard deviation (SD). Statistical significance was assessed using Student’s *t*-test, with a *p*-value <0.05 considered significant as previously described [26,69].

## 3. Results

### 3.1. Identification and Physicochemical Properties of the HECT Gene Family in N. benthamiana

In this study, an HMM was constructed based on the HECT protein sequences of six plant species: *A. thaliana*, *O. sativa*, *Z. mays*, *S. lycopersicum*, *G. max*, and *T. aestivum* (Table S1), and subsequently used to search against the whole-genome protein sequences of *N. benthamiana*. A total of 18 members of the HECT gene family were identified in *N. benthamiana* after the validation of conserved domains and the removal of redundant sequences. These genes were systematically designated as *NbHECT1* to *NbHECT18* according to their chromosomal locations (Table 1). An analysis of their physicochemical properties revealed that the genomic DNA sequences of the identified *NbHECT* genes ranged from 4235 to 56,645 bp in length, while the putative NbHECT proteins consisted of 588 to 3479 amino acid residues, with predicted molecular weights (MWs) ranging from 67.63 to 383.67 kDa. Notably, the isoelectric points (pIs) of the NbHECT proteins exhibited a slightly acidic characteristic, with 14 members having pI values below 7 (4.97–6.89), whereas only four members displayed weakly basic characteristics (7.21–8.56) (Table 1).

### 3.2. Phylogenetic Analysis of the NbHECT Gene Family

**Figure 1:**
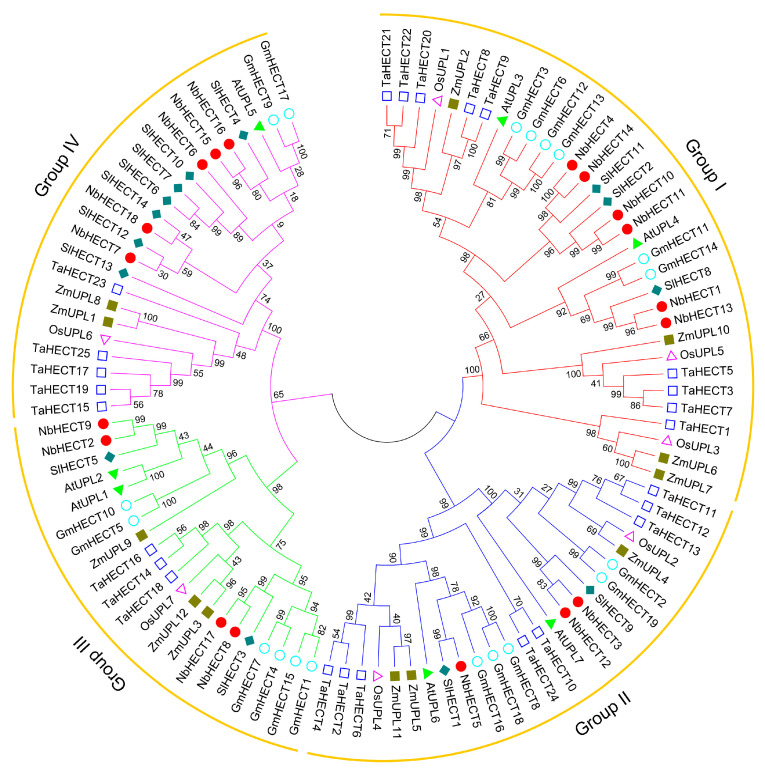
Phylogenetic analysis of the HECT-type E3 ubiquitin ligase (HECT) gene family from *N. benthamiana*, *A. thaliana*, *O. sativa*, *Z. mays*, *S. lycopersicum*, *G. max*, and *T. aestivum*. A phylogenetic tree was generated in MEGA 11.0 using the maximum-likelihood (ML) method, with branch support assessed by 1000 bootstrap replicates. HECTs from different plant species are represented by distinct colors, with red, blue, green, and pink clusters denoting Groups I, II, III, and IV, respectively.

To elucidate the evolutionary relationships among members of the NbHECT gene family, a phylogenetic tree was constructed using 18 *NbHECTs* in combination with 7 *A. thaliana ubiquitin-protein ligases* (*AtUPLs*)/*AtHECTs*, 7 *OsUPLs/OsHECTs*, 12 *ZmUPLs/ZmHECTs*, 14 *SlHECTs*, 19 *GmHECTs*, and 25 *TaHECTs* (Table S1). As shown in Figure 1, these 102 *HECTs* were classified into four subfamilies: Groups I, II, III, and IV. Group I, the largest subgroup, comprised 6 *NbHECT* members, 2 *AtHECT* members, 3 *OsHECT* members, 4 *ZmHECT* members, 3 *SlHECT* members, 6 *GmHECT* members, and 9 *TaHECT* members. Group II consisted of 3 *NbHECT* members, 2 *AtHECT* members, 2 *OsHECT* members, 3 *ZmHECT* members, 2 *SlHECT* members, 5 *GmHECT* members, and 8 *TaHECT* members. Group III possessed 4 *NbHECT* members, 2 *AtHECT* members, 1 *OsHECT* member, 3 *ZmHECT* members, 2 *SlHECT* members, 6 *GmHECT* members, and 3 *TaHECT* members, and therefore was the smallest subfamily. Group IV contained 5 *NbHECT* members, 1 *AtHECT* member, 1 *OsHECT* member, 2 *ZmHECT* members, 7 *SlHECT* members, 2 *GmHECT* members, and 5 *TaHECT* members (Figure 1). Notably, Groups I and IV were the subfamilies with the highest number of *NbHECTs* (Figure 1). These results indicate a more closely related evolutionary relationship between the *HECT* genes of *N. benthamiana* and *S. lycopersicum* compared to other analyzed taxa.

### 3.3. Gene Structure Analysis of the NbHECT Gene Family

**Figure 2:**
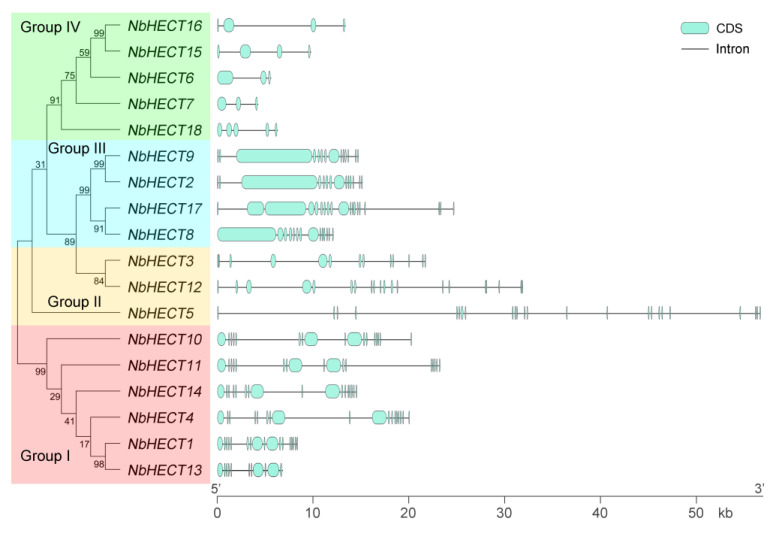
Schematic representation of the molecular phylogenetic relationships and gene structures of the *HECT-type E3 ubiquitin ligase* (*HECT*) genes in *N. benthamiana*. The unrooted phylogenetic tree was generated using the full-length NbHECT protein sequences with 1000 bootstrap replicates. The exon–intron architecture of *NbHECT* genes was analyzed using the Gene Structure Display Server 2.0 (GSDS 2.0). The exon and intron lengths for each *NbHECT* gene were proportionally represented to illustrate structural features.

To gain deeper insights into the structural diversity of *NbHECT* genes, we generated an ML phylogenetic tree and analyzed the exon–intron organization using the online tool GSDS 2.0 [49]. As shown in Figure 2, the number of exons exhibited considerable variation across different *HECT* genes. Among them, *NbHECT5* showed the most complex exon organization, comprising 24 exons, whereas *NbHECT6* and *NbHECT7* displayed the simplest architecture, with only three exons each (Figure 2). Interestingly, all *NbHECT* genes belonging to Groups I, II, and III exhibited gene architectures comprising more than 10 exons, whereas those in Group IV contained fewer than 10 exons (Figure 2). It is noteworthy that the majority of *NbHECT* genes in the same subfamily possessed the same number of exons, such as *NbHECT1*, *NbHECT10*, *NbHECT11*, and *NbHECT14* in Group I and *NbHECT2*, *NbHECT8*, and *NbHECT9* in Group III (Figure 2). These results indicate that phylogenetic clustering is correlated with structural conservation among *NbHECT* genes, suggesting shared functional characteristics and evolutionary trajectories within each clade.

### 3.4. Chromosomal Location and Synteny Analyses of the NbHECT Genes

**Figure 3:**
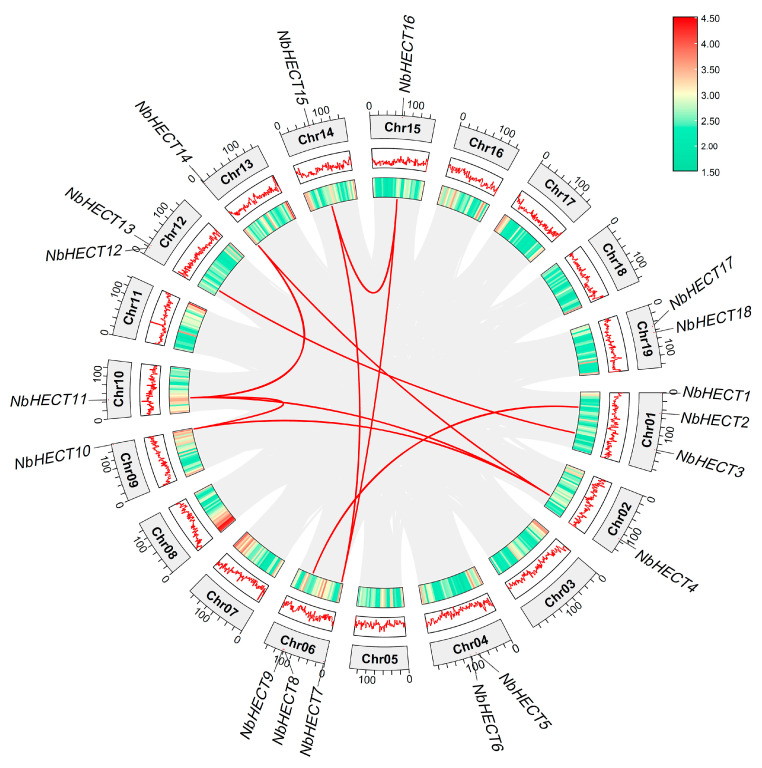
Schematic representation of the chromosomal distribution and duplication patterns of 18 *HECT-type E3 ubiquitin ligase* (*HECT*) genes in *N. benthamiana*. Red dots and black lines indicate the chromosomal locations of *NbHECT* genes, and the red lines represent duplicated gene pairs. Chromosome numbers are labeled within each respective chromosome. The scale bar indicates the gene density across the chromosomes of *N. benthamiana*.

To determine the chromosomal distribution of *NbHECT* genes in the *N. benthamiana* genome, we analyzed their chromosomal positions using the locus data obtained from the *N. benthamiana* Genome Project. As shown in Figure 3, a total of 18 *NbHECT* genes were unevenly distributed across 19 chromosomes. A single locus of genes was identified on chromosomes 02, 09, 10, 13, 14, and 15; chromosomes 04, 12, and 19 each harbored two genes; chromosomes 01 and 06 each contained three genes; while chromosomes 03, 05, 07, 08, 11, 16, 17, and 18 did not contain any *NbHECT* genes (Figure 3). To obtain a more comprehensive understanding of the evolutionary duplication patterns of *NbHECT* genes, a collinearity analysis was conducted using their chromosomal positions. As a result, a total of 10 segmental duplication events and no tandem duplication events were identified in the *N. benthamiana* genome (Figure 3 and Table 2). This indicates that segmental duplication may act as a primary driving force in the evolution of *NbHECT* genes in *N. benthamiana*. To assess evolutionary divergence and selection pressures among these duplicated gene pairs, we calculated Ka and Ks substitution rates using TBtools-II (v2.148) [51]. As shown in Table 2, the Ka/ Ks ratios of 10 duplicated gene pairs were all below 1, indicating that the *HECT* gene family in *N. benthamiana* has predominantly undergone purifying selection. Furthermore, the evolutionary dates of these duplication events were estimated using the formula T = Ks/2λ, which has been previously established for *N. benthamiana* [56]. These results suggest that 10 segmental duplications likely occurred between 1.75 and 21.38 million years ago.

### 3.5. Promoter Analysis of the NbHECT Genes

**Figure 4:**
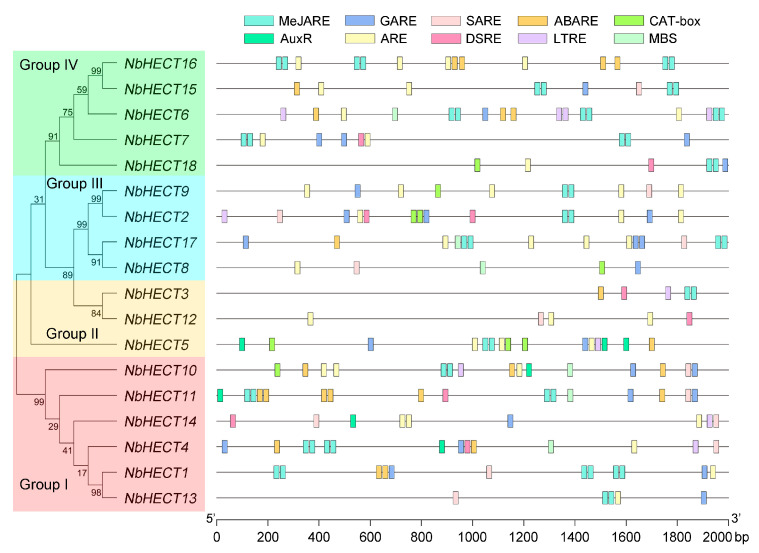
Schematic representation of the molecular phylogenetic relationships and 2000 bp promoters of the *HECT-type E3 ubiquitin ligase* (*HECT*) genes in *N. benthamiana*. The unrooted phylogenetic tree was generated using the full-length NbHECT protein sequences with 1000 bootstrap replicates. The 2000 bp promoter sequences of *HECT* genes were analyzed using PlantCARE and visualized using TBtools-II (v2.148). Different colored boxes represent different *cis*-acting elements.

To further elucidate the potential functions and regulatory mechanisms of *NbHECTs*, we performed a prediction and analysis of *cis*-acting elements in the promoter regions of *NbHECT* genes using the PlantCARE database [50]. The results revealed a large number of phytohormone- and growth-related regulatory elements in the regulatory regions of the *NbHECT* genes. These included the MeJA-responsive element (MeJARE), gibberellin-responsive element (GARE), salicylic acid (SA)-responsive element (SARE), abscisic acid (ABA)-responsive element (ABARE), meristem expression element (CAT-box), and auxin-responsive element (AuxRE) (Figure 4). Among the 18 *NbHECT* genes, 15 harbored the MeJARE element, 15 contained the GARE element, 12 possessed the SARE element, 10 included the ABARE element, 6 possessed the CAT-box element, and 5 contained the AuxRE element (Figure 4 and Table S4). In addition to the phytohormone- and growth-related regulatory elements, a considerable number of stress-responsive regulatory elements were also identified within the promoter regions of *NbHECT* genes (Figure 4). These included the anaerobic response element (ARE), defense- and stress-responsive element (DSRE), low-temperature-responsive element (LTRE), and MYB binding site (MBS). Of the 18 *NbHECT* genes, 16 contained the ARE element, 8 harbored the DSRE element, 7 contained the LTRE element, and 6 possessed the MBS element (Figure 4 and Table S4). These findings indicate that the promoters of *NbHECT* genes contain diverse *cis*-acting elements related to phytohormone signaling, growth processes, and stress responses, suggesting that *NbHECTs* may play crucial roles in modulating multiple phytohormone pathways, developmental programs, and plant responses to environmental stresses.

### 3.6. Tissue-Specific Expression Patterns of the NbHECT Genes

**Figure 5:**
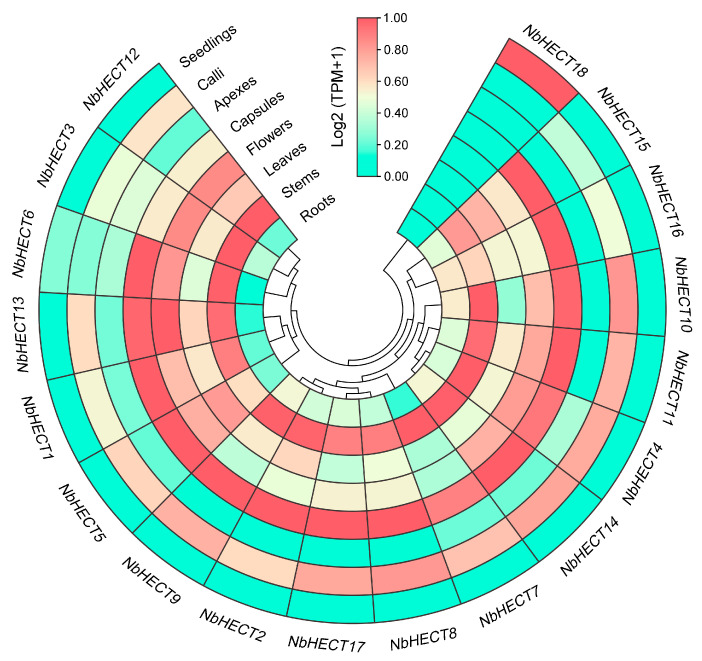
Expression profiles of the *HECT-type E3 ubiquitin ligase* (*HECT*) genes in *N. benthamiana*. The data used for tissue expression were obtained from the NCBI sequence read archive (SRA) database under the accession number PRJNA188486 [57], and the expression level of each gene is colored based on its Log2 (TPM + 1) values calculated from eight tissues: roots, stems, leaves, flowers, capsules, apices, calli, and seedlings. The heatmap was produced using TBtools-II (v2.148).

To elucidate the spatiotemporal expression patterns of *NbHECT* genes, we analyzed their transcript abundance across eight distinct tissue types of *N. benthamiana* using published RNA-sequencing datasets under the BioProject accession number PRJNA188486 [57]. The results indicated that all 18 *NbHECT* genes exhibited expression in at least one tissue (Figure 5). Strikingly, 17 of them (*NbHECT1*–*NbHECT17*) showed constitutive expression patterns across all examined tissues, with TPM values greater than 1.0 (TPM > 1.0) (Figure 5 and Table S5). Interestingly, all *NbHECT* genes (*NbHECT2*, *NbHECT8*, *NbHECT9*, and *NbHECT17*) in Group III exhibited high expression levels (TPM ≥ 5.0), suggesting that this subfamily may play a crucial role in regulating the growth and development of *N. benthamiana* (Figure 5 and Table S5). Furthermore, 11 genes, including *NbHECT1*, *NbHECT2*, *NbHECT4*, *NbHECT6*, *NbHECT8*–*NbHECT11*, *NbHECT14*, *NbHECT15*, and *NbHECT17*, displayed high expression levels in the stems and capsules of *N. benthamiana* (TPM ≥ 15.0). Among them, *NbHECT11* showed the highest expression in the stems (TPM ≥ 42.0), while *NbHECT17* exhibited the highest expression in the capsules (TPM ≥ 48.0) (Figure 5 and Table S5). Seven genes, namely, *NbHECT2*, *NbHECT8*, *NbHECT10*, *NbHECT11*, *NbHECT14*, *NbHECT15*, and *NbHECT17*, displayed high expression levels in the roots, leaves, flowers, and calli of *N. benthamiana* (TPM ≥ 15.0), with the highest expression levels observed for *NbHECT17* in the roots, capsules, and calli (TPM ≥ 20.0), and *NbHECT11* in the stems, leaves, and flowers (TPM ≥ 20.0), respectively (Figure 5 and Table S5). These results indicate that *NbHECT* genes exhibit distinct tissue-specific expression patterns, suggesting their potential roles in modulating diverse biological processes in *N. benthamiana*.

### 3.7. Expression Profiles of NbHECT Genes in Response to TYLCV Infection

**Figure 6:**
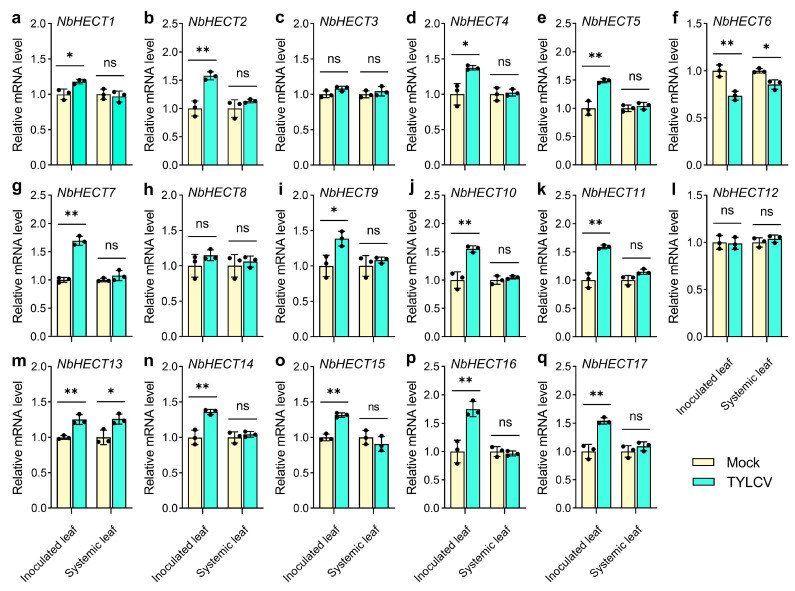
Expression levels of the *HECT-type E3 ubiquitin ligase* (*HECT*) genes in *N. benthamiana* in response to tomato yellow leaf curl virus (TYLCV) infection. (**a**–**q**) Relative expression levels of *NbHECT1*–*NbHECT17* in inoculated and systemically infected leaves of *N. benthamiana* following TYLCV infection. The data represent relative mRNA expression levels in leaves infected with *Agrobacterium tumefaciens* carrying an empty vector (Mock), which were normalized to a reference value of 1.0. Results are presented as the mean ± standard deviation from three independent biological replicates. Statistically significant differences are indicated by asterisks: * *p* < 0.05 or ** *p* < 0.01; ns, not significant (Student’s *t*-test).

To elucidate the potential roles of *NbHECT* genes in TYLCV defense responses, we selected *NbHECTs* with basal expression levels in the leaves of *N. benthamiana* (TPM ≥1.0) (Table S5) and examined their expression profiles following TYLCV infection using qPCR. The results showed that the expression of *NbHECT1*–*NbHECT5*, *NbHECT7*, *NbHECT9*–*NbHECT11*, and *NbHECT13*–*NbHECT17* was significantly upregulated in the inoculated leaves of *N. benthamiana* following TYLCV infection, while *NbHECT6* exhibited a pronounced downregulation pattern (Figure 6). In the systemically infected leaves, only two genes, *NbHECT6* and *NbHECT13*, displayed downregulated and upregulated patterns, respectively, following TYLCV infection (Figure 6). However, due to the exceptionally low basal expression level of *NbHECT18* in all examined tissues (Figure 5 and Table S5), its expression was not detected in either the inoculated leaves or the systemically infected leaves. These results suggest that *NbHECTs* are likely to play a key role in the early defense response to TYLCV infection, particularly *NbHECT6* and *NbHECT13*, which exhibit a consistent expression pattern in both inoculated and systemic leaves.

### 3.8. Disruption of the Expression of NbHECTs Increases Host Susceptibility to TYLCV

**Figure 7:**
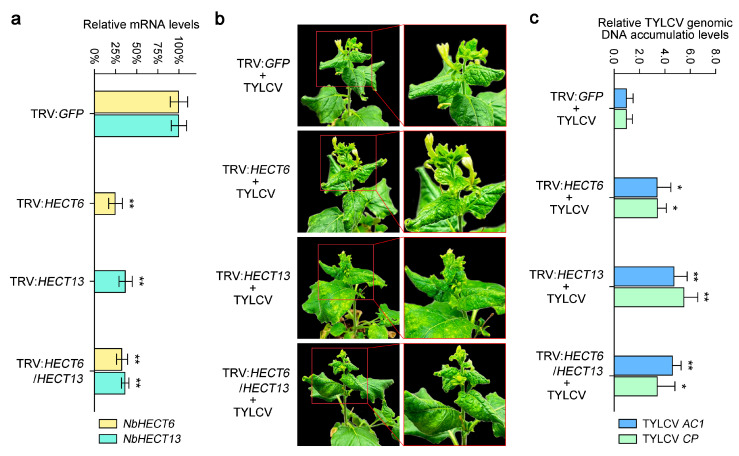
Silencing the *NbHECT6* and *NbHECT13* genes in *N. benthamiana* increases its susceptibility to tomato yellow leaf curl virus (TYLCV) infection. (**a**) Silencing efficiency was evaluated by quantitative PCR (qPCR) at 10 days post-inoculation (dpi). The data are expressed as relative mRNA levels, with values for the control group (*N. benthamiana* plants agroinfiltrated with TRV:GFP) set at 100%. (**b**) Disease symptoms induced by TYLCV in *NbHECT6* and *NbHECT13* silenced *N. benthamiana* plants at 21 dpi. TRV:GFP agroinfiltrated *N. benthamiana* seedlings infected with TYLCV served as the control. (**c**) Relative accumulation of TYLCV genomic DNA was assessed in the *NbHECT6* and *NbHECT13* silenced *N. benthamiana* plants. Viral accumulation was quantified by qPCR, as depicted in (**a**). Data are presented as relative viral DNA accumulation, with values for the TRV:GFP control plants set to 1.0. For panels (**a**,**c**), data are presented as means ± standard deviation of three independent biological replicates. Statistically significant differences are indicated by asterisks: * *p* < 0.05 or ** *p* < 0.01, as determined by Student’s *t*-test.

To further clarify the functional roles of *NbHECTs* in the response to TYLCV infection, we employed the VIGS system to investigate their specific involvement during viral infection. According to the tissue-specific expression patterns and gene expression profiles of *NbHECTs* following TYLCV infection (Figure 5 and Figure 6), two genes, *NbHECT6* and *NbHECT13*, were selected for individual or together silencing using a TRV-mediated VIGS approach [67]. Compared with the vector control (*N. benthamiana* agroinfiltrated with TRV:GFP), the mRNA levels of *NbHECT6* and *NbHECT13* in plants infiltrated with the silencing constructs were reduced by about 60–80% at 10 dpi (Figure 7a), confirming successful silencing of the target genes through VIGS. Following VIGS treatment, both control and gene-silenced plants were inoculated with an infectious clone of TYLCV and systematically monitored over time. At 21 dpi, compared to the TRV:GFP control plants, systemic leaves of *NbHECT6*- and *NbHECT13*-silenced plants exhibited severe curling and wrinkling symptoms induced by TYLCV (Figure 7b). These findings indicate that the suppression of *NbHECT6* and *NbHECT13* enhances the susceptibility of *N. benthamiana* to TYLCV infection. To further confirm these observations, the accumulation of TYLCV genomic DNA was assessed by determining the expression levels of TYLCV *AC1* and *CP* genes via qPCR, following established protocols [26,70]. The results revealed that silencing of *NbHECT6* and *NbHECT13* in *N. benthamiana* plants led to an approximately three-fold increase in TYLCV genomic DNA accumulation relative to control plants (Figure 7c). Interestingly, viral DNA accumulation in the double gene-silenced plants was found to be intermediate between that observed in the two single gene-silenced groups (Figure 7c). Collectively, these results suggest that the suppression of *NbHECT6* and *NbHECT13* significantly compromises the resistance of *N. benthamiana* to TYLCV, as indicated by a marked increase in viral genomic DNA accumulation in host tissues.

## 4. Discussion

Previous studies have shown that the UPS plays a central role in regulating plant growth, development, and stress responses by controlling protein turnover and signaling cascades [5,6,7,8]. In this study, we systematically identified and characterized 18 *NbHECTs* in *N. benthamiana*, providing new insights into their structural diversity, evolutionary conservation, and functional roles in antiviral defense. Phylogenetic analysis demonstrated that *NbHECTs* clustered into four distinct subfamilies (Groups I–IV) (Figure 1), consistent with the classification observed in *S. lycopersicum* and *A. thaliana* [14,39], suggesting an ancient evolutionary origin of this gene family in plants. Notably, the closer evolutionary relationship between HECT genes in *N. benthamiana* and *S. lycopersicum*, as compared to those in other species (Figure 1), may reflect their shared susceptibility to viral pathogens such as TYLCV [22,23,24,25]. This phylogenetic conservation implies that *NbHECTs* may have retained critical regulatory functions in *Solanaceae*-specific stress responses.

Structural analysis revealed that *NbHECT* genes exhibit significant variation in exon–intron organization, with exon numbers ranging from 3 (*NbHECT6* and *NbHECT7*) to 24 (*NbHECT5*) (Figure 2 and Table 1). This structural diversity likely reflects functional specialization, as genes within the same phylogenetic group maintained conserved exon numbers (e.g., Group I and III members). Such conservation suggests strong selective pressures to maintain gene architecture, possibly due to constraints on protein domain organization or regulatory elements [16,17,20]. The predominance of segmental duplication events (10 detected) over tandem duplications (Figure 3 and Table 2) suggests that whole-genome duplications rather than localized gene amplifications drove NbHECT family expansion. This finding is consistent with the gene duplication patterns observed in HECT gene families across other plant species [42,43]. The estimated divergence times (1.75–21.38 million years ago) coincide with periods of *Solanaceae* diversification [53,54,71], further supporting their role in adaptive evolution. Furthermore, the Ka/Ks ratios (<1) across all duplicated pairs (Table 2) suggest purifying selection has maintained critical functional divergence, particularly the catalytic HECT domain required for Ub transfer [15,16,17].

Promoter analysis revealed the presence of numerous *cis*-acting regulatory elements associated with phytohormone signaling and stress responses (Figure 4 and Table S4), indicating that *NbHECTs* may serve as integrators of multiple signaling pathways. The high prevalence of MeJAREs and GAREs (15 out of 18 genes) is particularly notable, given the critical role of jasmonate and gibberellin signaling pathways in antiviral defense [72,73,74]. Additionally, the presence of stress-responsive elements (AREs, DSREs, and LTREs) in most *NbHECT* promoters further supports their involvement in abiotic and biotic stress responses (Figure 4 and Table S4). These findings align with studies in *A. thaliana*, *Malus domestica*, *Brassica rapa*, *B. oleracea*, and *Phyllostachys edulis*, where HECTs modulate hormone crosstalk and stress responses [39,75,76,77], suggesting a conserved regulatory network across plant species. Tissue-specific expression analysis demonstrated that 17 out of 18 *NbHECTs* are constitutively expressed (TPM > 1.0) across all examined tissues (Figure 5 and Table S5), consistent with their putative roles in fundamental cellular functions, including protein homeostasis and signal transduction pathways [6,9]. Notably, Group III members (*NbHECT2*, *NbHECT8*, *NbHECT9*, and *NbHECT17*) exhibited the highest expression levels in reproductive tissues (TPM ≥ 5.0), suggesting their specialized functions in reproductive development or stress responses [20,78,79]. Furthermore, the strong expression of *NbHECT6* and *NbHECT13* in leaves (Figure 5) and their differential regulation during TYLCV infection (Figure 6) further highlights their potential role in leaf-specific antiviral responses.

It has been shown that an HECT domain-containing protein, Rsp5p, can bind to the p33 and p92 proteins encoded by tomato bushy stunt virus and inhibit its replication in yeast [80]. In *S. lycopersicum* and *N. benthamiana*, plant HECT proteins have also been reported to participate in antiviral responses [14,81]. In this study, it was found that *NbHECTs* showed a differential expression pattern during TYLCV infection (Figure 6), providing direct evidence for their involvement in antiviral responses. Although most *NbHECTs* were upregulated in inoculated leaves (Figure 6), the consistent downregulation of *NbHECT6* in both local and systemic infections suggests TYLCV may actively suppress this gene to evade host defenses, similar to how geminiviral C2 protein interferes with ubiquitination [82,83]. Conversely, the upregulation of *NbHECT13* suggests a potentially positive role in antiviral signaling, which may be mediated through the ubiquitination and subsequent degradation of viral proteins or via the modulation of defense-related hormonal pathways [84,85]. Functional validation through VIGS revealed that the silencing of *NbHECT6* and *NbHECT13* aggravates TYLCV symptoms (Figure 7b) and leads to approximately a threefold increase in viral DNA accumulation (Figure 7c). These phenotypes are consistent with the effects observed upon the disruption of UPS components in other plant–virus systems [86,87], supporting an evolutionarily conserved role for E3 ligases in antiviral defense. These findings align with emerging models of Ub-mediated antiviral defense in higher plants [88,89]. Although the exact roles of *NbHECTs* in the response of *N. benthamiana* to TYLCV infection remain to be fully elucidated, our findings enhance the understanding of the expansion of the NbHECT gene family and offer valuable insights into the functional characterization of these genes in mediating resistance against TYLCV.

## 5. Conclusions

In this study, we conducted the first comprehensive analysis of the HECT gene family in *N. benthamiana* through gene identification, phylogenetic analysis, gene structure characterization, promoter analysis, and tissue-specific and TYLCV-responsive expression profiling. A total of 18 *NbHECT* genes were identified across the entire genome, providing valuable insights for the functional characterization of the HECT gene family in *N. benthamiana*. Furthermore, our VIGS and qPCR data revealed that TYLCV genomic DNA accumulation was significantly enhanced upon silencing of the two *NbHECTs* (*NbHECT6* and *NbHECT13*) in *N. benthamiana*. These findings contribute to a better understanding of the molecular processes mediated by the *NbHECT* genes in response to TYLCV infection and lay the groundwork for a more systematic exploration of the functional mechanisms of the HECT gene family in *N. benthamiana*.

## Figures and Tables

**Table 1:** Structural and physicochemical characterization of the HECT-type E3 ubiquitin ligase (HECT) gene family in *N. benthamiana*.

Gene Name	Gene ID ^a^	Gene Position	Strand	Size					
				Genomic DNA (bp)	Number of Exons	CDS ^b^ (bp)	Protein (aa)	MW ^c^ (kDa)	pI ^d^
*NbHECT1*	Niben261Chr01g0009001	Niben261Chr01:939808–948152	forward	8345	17	4767	1588	176.63	5.52
*NbHECT2*	Niben261Chr01g0481001	Niben261Chr01:48077477–48092623	forward	15,147	14	10,248	3415	373.11	5.03
*NbHECT3*	Niben261Chr01g1345011	Niben261Chr01:134516798–134538509	forward	21,712	13	3354	1117	126.60	8.56
*NbHECT4*	Niben261Chr02g1146005	Niben261Chr02:114614528–114634495	forward	19,968	18	5508	1835	198.99	5.84
*NbHECT5*	Niben261Chr04g0871004	Niben261Chr04:87053772–87110416	forward	56,645	24	2649	882	100.56	5.51
*NbHECT6*	Niben261Chr04g1019003	Niben261Chr04:101911370–101916954	forward	5585	3	2577	858	98.89	6.68
*NbHECT7*	Niben261Chr06g0028002	Niben261Chr06:2825647–2829881	reverse	4235	3	1767	588	67.63	8.08
*NbHECT8*	Niben261Chr06g1000013	Niben261Chr06:100017780–100029875	forward	12,096	14	10,179	3392	374.42	4.97
*NbHECT9*	Niben261Chr06g1026001	Niben261Chr06:102542575–102557284	forward	14,710	14	10,248	3415	373.07	5.00
*NbHECT10*	Niben261Chr09g1363007	Niben261Chr09:136341123–136361305	forward	20,183	17	5577	1858	199.45	5.69
*NbHECT11*	Niben261Chr10g0448001	Niben261Chr10:44821858–44845061	reverse	23,204	17	5634	1877	201.67	5.62
*NbHECT12*	Niben261Chr12g0194001	Niben261Chr12:19384125–19415980	forward	31,856	20	4194	1397	157.50	8.27
*NbHECT13*	Niben261Chr12g0254002	Niben261Chr12:25391951–25398716	reverse	6766	11	4104	1367	151.86	5.54
*NbHECT14*	Niben261Chr13g0020011	Niben261Chr13:2046409–2060925	reverse	14,517	17	5520	1839	198.30	5.77
*NbHECT15*	Niben261Chr14g0693017	Niben261Chr14:69254130–69263876	reverse	9747	4	2286	761	86.53	5.94
*NbHECT16*	Niben261Chr15g0756024	Niben261Chr15:75656777–75670163	reverse	13,387	4	2133	710	81.42	5.62
*NbHECT17*	Niben261Chr19g0507029	Niben261Chr19:50718936–50743619	reverse	24,684	19	10,440	3479	383.67	5.05
*NbHECT18*	Niben261Chr19g0656003	Niben261Chr19:65653994–65660273	forward	6280	5	2280	759	85.83	7.48

^a^ Gene ID, the gene locus in the *N. benthamiana* Genome Project (https://solgenomics.net/organism/Nicotiana_benthamiana/genome/ (accessed on 2 December 2023)). ^b^ CDS, coding sequence. ^c^ MW, molecular weight. ^d^ pI, isoelectric point.

**Table 2:** Divergence analysis of duplicated *HECT-type E3 ubiquitin ligase* (*HECT*) gene pairs in *N. benthamiana*.

Gene Pairs		Ka ^a^	Ks ^b^	Ka/Ks	Duplicate Type	Purifying Selection ^c^	Duplication Date (MY ^d^)
*NbHECT2*	*NbHECT9*	0.02	0.05	0.31	Segmental	Yes	1.75
*NbHECT3*	*NbHECT12*	0.03	0.08	0.38	Segmental	Yes	2.59
*NbHECT4*	*NbHECT10*	0.11	0.57	0.20	Segmental	Yes	18.91
*NbHECT4*	*NbHECT11*	0.11	0.55	0.20	Segmental	Yes	18.23
*NbHECT4*	*NbHECT14*	0.03	0.06	0.41	Segmental	Yes	2.16
*NbHECT7*	*NbHECT15*	0.20	0.63	0.32	Segmental	Yes	20.99
*NbHECT7*	*NbHECT16*	0.22	0.64	0.34	Segmental	Yes	21.38
*NbHECT10*	*NbHECT11*	0.01	0.07	0.20	Segmental	Yes	2.33
*NbHECT11*	*NbHECT14*	0.10	0.55	0.19	Segmental	Yes	18.45
*NbHECT15*	*NbHECT16*	0.01	0.09	0.11	Segmental	Yes	3.06

^a^ Ka, Non-synonymous substitution rate. ^b^ Ks, Synonymous substitution rate. ^c^ Ka/Ks ratio < 1 is considered purifying selection for plant *HECTs*. ^d^ MY, Million years.

## Data Availability

All data generated or analyzed during this study have been included in this article and its Supplementary Materials.

## Supplementary Materials

The following supporting information can be downloaded at: https://www.mdpi.com/article/10.3390/genes16101150/s1, Table S1: Protein sequences of HECT E3 ligases of *Arabidopsis thaliana*, *Oryza sativa*, *Zea mays*, *Solanum lycopersicum*, *Glycine max*, and *Triticum aestivum*, and *Nicotiana benthamiana*; Table S2: List of primers used in this study; Table S3: Raw data used for qPCR analysis of *NbHECT* genes in response to TYLCV infection; Table S4: *Cis*-acting elements in the promoter regions of *NbHECT* genes in *Nicotiana benthamiana*; Table S5: Tissue-specific expression profiles of *NbHECT* genes in *Nicotiana benthamiana*.
